# New Opportunities for High‐Performance Source‐Gated Transistors Using Unconventional Materials

**DOI:** 10.1002/advs.202101473

**Published:** 2021-08-27

**Authors:** Gang Wang, Xinming Zhuang, Wei Huang, Junsheng Yu, Huaiwu Zhang, Antonio Facchetti, Tobin J. Marks

**Affiliations:** ^1^ State Key Laboratory of Electronic Thin Films and Integrated Devices University of Electronic Science and Technology of China Chengdu 610054 P. R. China; ^2^ Department of Chemistry and the Materials Research Center Northwestern University 2145 Sheridan Road Evanston IL 60208 USA; ^3^ School of Physics State Key Laboratory of Crystal Materials Shandong University Jinan 250100 P. R. China; ^4^ School of Automation Engineering University of Electronic Science and Technology of China (UESTC) Chengdu Sichuan 611731 P. R. China; ^5^ Flexterra Corporation Skokie IL 60077 USA

**Keywords:** dielectric, energy efficient transistors, source gated transistors, transistors, unconventional transistor materials

## Abstract

Source‐gated transistors (SGTs), which are typically realized by introducing a source barrier in staggered thin‐film transistors (TFTs), exhibit many advantages over conventional TFTs, including ultrahigh gain, lower power consumption, higher bias stress stability, immunity to short‐channel effects, and greater tolerance to geometric variations. These properties make SGTs promising candidates for readily fabricated displays, biomedical sensors, and wearable electronics for the Internet of Things, where low power dissipation, high performance, and efficient, low‐cost manufacturability are essential. In this review, the general aspects of SGT structure, fabrication, and operation mechanisms are first discussed, followed by a detailed property comparison with conventional TFTs. Next, advances in high‐performance SGTs based on silicon are first discussed, followed by recent advances in emerging metal oxides, organic semiconductors, and 2D materials, which are individually discussed, followed by promising applications that can be uniquely realized by SGTs and their circuitry. Lastly, this review concludes with challenges and outlook overview.

## Graphical abstract

The unique device architecture and intrinsically different operating mechanism enable source‐gated transistors (SGTs) to achieve ultrahigh gain, lower power consumption, lower saturation voltage, higher bias stress stability, immunity to short‐channel effects, and greater tolerance to geometric variations. These characteristics make SGTs ideal candidates for readily fabricated displays, biomedical sensors, integrated analog and digital circuits, and wearable electronics.

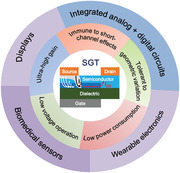

## 1 Introduction

The transistor is one of the basic building blocks in modern electronics, has been widely adopted in integrated circuits, and enables the regulation and amplification of electronic signals.^[^ ^1^ ^]^ Thin film transistors (TFTs), as one class of transistor, were first reported by Weimer in 1962.^[^ ^2^ ^]^ TFTs based on amorphous silicon, polysilicon, and indium gallium zinc oxide have now become relatively mature materials‐based technologies, especially in the area of flat panel displays.^[^ ^3^ ^]^ Despite the great commercial success of these TFT technologies, novel semiconducting materials, new device modalities, and new fabrication techniques to satisfy the demands for higher performance, lower manufacturing costs, and multiple functionalities will be required for next‐generation electronics, including portable/wearable electronics, flexible/stretchable electronics, and transparent electronics for communication, control, display, environmental sensing, and biomedical applications.^[^ ^3^, ^4^ ^]^ For these reasons, emerging semiconductor classes, including metal oxides, transition metal dichalcogenides, and organic materials have garnered intense research interest.^[^ ^5^, ^6^ ^]^ Metal oxide semiconductors, especially amorphous oxides semiconductors, have been extensively investigated owing to their optical transparency, high electron mobility, remarkable environmental/thermal stability, superior mechanical flexibility, low processing temperatures, and high morphological uniformity compared to conventional silicon.^[^ ^7^, ^8^, ^9^, ^10^, ^11^ ^]^ Concurrently, organic semiconductors have contributed greatly to the development of low‐power and flexible electronics, especially for displays as well as wearable and biocompatible technologies.^[^ ^12^, ^13^, ^14^, ^15^, ^16^, ^17^ ^]^ Moreover, to reduce fabrication costs, solution processing of the TFT components by, e.g., spin coating, spray coating and printing, has attracted considerable attention from both the research and industrial communities, and could provide access to inexpensive devices with lower performance than poly‐Si and other inorganic semiconductors but targeting the supply chain, item identification, sensor, and e‐reader marketspaces.^[^ ^18^, ^19^, ^20^, ^21^, ^22^, ^23^ ^]^

Innovative device engineering, such as implementing heterojunction and interface modification, have also contributed to the enormous advances in TFT performance.^[^ ^24^, ^25^, ^26^, ^27^ ^]^ In recent years, emerging applications, such as wearable electronics and robotics will require devices with low power dissipation due to severe limitations in battery capacity. Common methods to reduce energy consumption focus on the adoption of high‐*κ* dielectrics, which can effectively achieve low driving voltages and low power operation.^[^ ^28^, ^29^, ^30^ ^]^ Additionally, electrolyte dielectrics have also been deployed to realize low operation voltages since capacitance > 1 µF cm^−2^ can be obtained via the formation of electric double layers; however, they bring some limitations, such as doping effects and delamination.^[^ ^31^, ^32^ ^]^ These approaches may also introduce undesirable characteristics such as large gate leakage currents, poor thermal/environmental/chemical stability, slow operating speeds, and incompatibility with the traditional Si production lines used in industry.

An alternative strategy to attain low power dissipation is by adopting source‐gated transistors (SGTs), which represent an extremely promising architecture due to an intrinsically different operating mechanism and performance characteristics compared to TFTs.^[^ ^33^, ^34^, ^35^, ^36^ ^]^ By creating a Schottky source barrier between the source contact and the semiconductor, the drain current is dominated by the source barrier height rather than by the accumulated charge carriers in the TFT channel. This materials combination allows SGTs to achieve low power consumption,^[^ ^37^, ^38^ ^]^ high intrinsic gain,^[^ ^39^, ^40^ ^]^ high thermal/environmental/chemical stability,^[^ ^41^ ^]^ reduced short‐channel effects,^[^ ^42^ ^]^ and low saturation voltages.^[^ ^43^ ^]^ While source‐gated effects were first applied in semiconducting hydrogenated amorphous silicon (a‐Si:H) devices, the recent emergence of high‐performance unconventional semiconducting materials such as oxides, organics, and 2D materials, when combined with the aforementioned attractions of SGT architectures, offer new opportunities for low‐power flexible/stretchable and/or optically transparent electronics, readily manufactured by solution/printing technologies. Displays, wearables, biomedical sensors, and the Internet of Things are among the applications possible for SGTs with new materials.^[^ ^44^, ^45^, ^46^, ^47^, ^48^, ^49^ ^]^ However, up until now, no review focusing on the developments and prospects for new SGT materials has appeared.

In this review, we discuss the most recent advances in source‐gated transistors from the aspects of device architecture, operation, and enabling materials. First, we overview the device structures and operating mechanism as well as attractions of SGTs versus conventional TFTs. Next, a discussion of high‐performance devices achieved by three specific classes of channel materials, oxides, 2D, and organics is presented, followed by a discussion of SGT applications. Finally, a conclusion and outlook section focuses on current challenges and future potential applications if major technical hurdles are surmounted.

## 2 Basic Structure and Operation of SGTs

**Figure 1:**
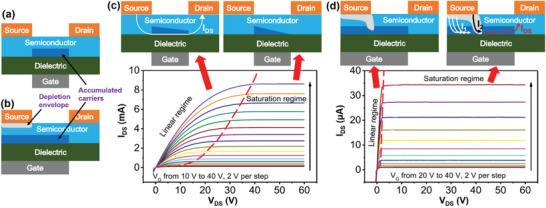
Schematic of a) conventional thin‐film transistor (TFT) and b) source‐gated transistor (SGT), where the depletion envelope and accumulated carrier layer are illustrated. Schematic and output characteristics of c) TFT and d) SGT, indicating the evolution of carrier accumulation and depletion region envelope from linear regime to saturation regime. Reproduced with permission.^[^ ^72^ ^]^ Copyright 2019, National Academy of Sciences.

Conventional SGTs are a combination of TFT with another fundamental component—a source barrier.^[^ ^50^ ^]^ In a conventional high‐performance TFTs (**Figure** **1**a), the contacts between the semiconductor and source/drain electrodes are ohmic. By applying a gate voltage (*V*_GS_), electron or hole carriers are accumulated and confined in the semiconductor layer at the interface with the gate dielectric, leading to the formation of a conductive channel. Upon applying a bias between the source and the drain (*V*_DS_), the accumulated carriers drift from source to drain electrodes, and generate the drain current (I_D_).^[^ ^51^ ^]^ I_D_ increases with increasing *V*_GS_ and *V*_DS_ until reaching saturation when *V*_DS_ is sufficiently large to pinch off the channel near the drain end. The process of how the current evolves with varying *V*_GS_ and *V*_DS_ in conventional TFT is illustrated in Figure 1c, where evolution of *I*_DS_ and carrier accumulation envelope from linear regime to saturation regime are demonstrated in output characteristics and device schematics. Note that Figure 1c,d shows electron charge transport from the source to the drain via arrows for n‐channel SGTs, which is opposite to the conventional current direction.

**Figure 2:**
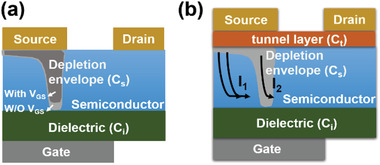
a) Schematic of a Schottky contact SGT. When there is no gate voltage, saturation will cause the semiconductor pinch‐off. By applying a gate voltage, due to the accumulation of charge carriers, the depletion region will be shallower. b) Tunnel‐contact SGT structure.

In SGTs, the materials are selected so as to create a potential barrier between the source contact and the semiconductor. The source barriers can be achieved by several approaches, the most common of which is to introduce a Schottky barrier and an insulator layer between the metal and the semiconductor layer.^[^ ^34^ ^]^ Since most of the SGTs are based on a Schottky barrier at the source contact, we first describe the basic mechanism of SGTs using this structure. As shown in Figure 1b, an SGT usually has a gate electrode that overlaps with the source and a Schottky barrier diode under the source, which operates in a reverse bias mode. During operation, with a certain *V*_GS_ bias, a depletion region under the source near the drain end will expand toward the semiconductor/dielectric interface upon applying a *V*_DS_ (Figure 1d). Before the depletion region reaches the semiconductor/dielectric interface, *I*_DS_ linearly increases along with *V*_DS_ for a given *V*_GS_ (linear regime in output characteristics). When the depletion region contacts the interface at a certain *V*_DS_, the source end of the channel is pinched off and *I*_DS_ saturates (Saturation regime). Hence, *I*_DS_ in SGTs is mainly controlled by source contact rather than the channel characteristics.^[^ ^48^ ^]^ Moreover, two distinct current injection mechanisms contribute to the overall drain current in SGTs (Figure 1d): 1) The current is injected from the bulk of the source electrode (*I*_1_). *I*_1_ depends on a nonlinear contact resistance due to the potential along the accumulation layer under the source,^[^ ^52^ ^]^ where *I*_DS_ is limited by the parasitic resistance effects of the high resistivity region near the source. 2) The current is injected from the source barrier in the high‐field pinch‐off region under the source (*I*_2_). The pinch‐off region at the edge of the source allows the gate‐induced electric field to reach the metal‐semiconductor contact, and thus modulate its reverse current *I*_2_. Depending on the magnitude of the electric field, either *I*_1_ (low‐field mode) or *I*_2_ (high‐field mode) dominates *I*_DS._^[^ ^53^, ^54^ ^]^ In the low‐field mode, the depletion region behaves rather like a junction gate thin‐film transistor, where the charge is located at the semiconductor‐dielectric interface. Furthermore, *I*_1_ has a uniform variable quantity as the gate voltage is varied. In this case, the source barrier height is not strongly correlated with the field intensity. However, at high fields, the source barrier lowering is proportional to electric field and the *I*_2_ is sensitive to changes in the source barrier height. Hence, *I*_2_ will depend on the drain voltage and its respective electric field. If there is no gate voltage, charge carriers must surmount a relatively large Schottky barrier by thermionic emission. However, when *V*_GS_ is applied, the charge carriers overcome the Schottky barrier under the thermionic‐field emission model. The application of a *V*_GS_ (positive for an n‐type and negative for a p‐type SGT) plays the role of an inverse voltage to the built‐in electric field in the depleted region, modulating the extent to which the depletion region penetrates the semiconductor. This modification of the depletion envelope only requires a small increase of *V*_DS_ to re‐establish the fully depleted semiconductor (**Figure** **2**a). The currents *I*_1_ and *I*_2_ in the depleted region under the source can then be described by the equation for a reverse bias Schottky diode according to Equations (1) and (2)^[^ ^54^, ^55^ ^]^

(1) $$I_{1}=SA*T^{2}\exp\left(-\frac{q\phi_{bh}}{kT}\right)$$

(2) $$I_{2}=SA*T^{2}\exp\left(-\frac{q\phi_{bh}-\alpha E_{S}}{kT}\right)$$

where *S* is the contact area, *A* is the Richardson's constant, *T* is the temperature, *Φ*_bh_ is the barrier height in the absence of interfacial anomalies, *α* is a tunneling constant, *E*_S_ (or *E*_dep_) is the electric field in the depletion region, and *k* is Boltzmann's constant. Furthermore, in high field mode, by measuring the saturation current at different temperatures and plotting the graph of ln(*I*_DS_/T^2^) versus *q*/*kT*, the effective Schottky‐barrier height as a function of *V*_GS_ can be determined. In contrast to TFTs where the off‐current (*I*_off_) relies on the intrinsic semiconductor conductance, SGTs can be turned off even if a conductor is acting as the channel, just by ensuring that the source contact is reversely biased and charge injection is prohibited. The potential at the semiconductor interface is then reversed with respect to the source, prohibiting the extraction of charges from the source. As a result, the device is turned off. Consequently, the current in an SGT is significantly lower due to the high contact resistance between source and semiconductor, and it usually has a significantly lower *I*_off_ than in TFTs.^[^ ^56^ ^]^

Furthermore, due to the facile carrier depletion of the semiconductor in a reverse biased Schottky diode, the required voltage to fully turn‐off an SGT is considerably smaller than that in a conventional ohmic‐contact TFT. Moreover, thicker semiconductors usually require a larger *V*_DS_ to achieve full depletion of the semiconductor closest to the source edge. The saturation voltage *V*_DSAT_ in Schottky barrier based SGT can be expressed as Equation (3)^[^ ^55^ ^]^

(3) $$V_{\mathrm{DSAT}}=\frac{C_{i}\left(V_{\mathrm{GS}}-V_{T}\right)}{C_{i}+C_{s}}\;$$

where *C*_i_ and *C*_s_ represent capacitances (per unit area or unit length) of the gate insulator and the depleted semiconductor, respectively; *V*_T_ is threshold voltage. While in conventional ohmic‐contact TFTs, *V*_DSAT_ is determined as *V*_DSAT_ = *V*_GS_ − *V*_T_, but since *C*_s_ can be relatively large, *V*_DSAT_ in SGTs always shows a significantly smaller value than that in TFTs.

SGTs can also be fabricated by inserting an ultrathin insulating layer between the source contact and the semiconductor, which is called a tunnel‐contact SGT (Figure 2b). By using this architecture, an additional series capacitance (*C*_t_: capacitance of the tunneling layer) is introduced in between the pinch‐off point and the edge of the source contact. Thus, *V*_DSAT_ in this tunnel‐contact SGT is expressed by Equation (4)^[^ ^57^ ^]^

(4) $$V_{\mathrm{DSAT}}=\frac{C_{i}\left(V_{\mathrm{GS}}-V_{T}\right)}{C_{i}+C_{s}+C_{t}}\;$$

Note here that *C*_t_ is usually much greater than *C*_s_ due to ultrathin nature of this layer; hence, *V*_DSAT_ is mainly determined by the properties of the tunnel layer rather than those of the depleted semiconductor. The current in tunnel‐contact SGTs has working principles similar to those in Schottky contact SGTs. In low‐field modes, the ultrathin dielectric layer can tolerate significantly higher current densities and the tunnel layer effectively transmits I_1_ injected from the bulk of the source dependents on nonlinear contact resistance under the bulk source, similar to the Schottky contact SGT. However, at high fields, since *C*_s_ ≪ *C*_t_, only a small drop of *V*_DSAT_ exists across the tunneling diode, and the majority drop of *V*_DSAT_ is across the depleted semiconductor. Hence, *I*_2_ injected from the edge of the source into the depletion region dominates. Moreover, the ultrathin dielectric layer is responsible for the efficiency of charge transport across the contact, and as a result, the on‐current of tunnel‐contact SGTs is high and comparable to the level of TFTs due to the tunneling behavior. Furthermore, the metal‐insulator‐semiconductor barrier in tunnel‐contact SGTs can be engineered to provide tunneling at the Fermi level, so they could in principle obtain pure field emission with a nearly zero temperature coefficient. Therefore, such tunnel‐contact SGTs are predicted to have smaller temperature dependence of the current than Schottky‐contact SGTs.^[^ ^34^, ^57^ ^]^

Due to the unique working mechanism, SGTs hold many potential advantages over TFTs. Since *I*_DS_ is controlled by the dimensions of the source contact and barrier height rather than by the transistor channel, one major advantage is that the source contact rather than the channel determines the current, there is greater tolerance in the fabrication of the different SGT layers. Thus, no precise registration and resolution is required in SGT fabrication process. Therefore, SGTs are far more immune to short‐channel effects and channel length variation. Due to large impedance and low *V*_DSAT_, another advantage of SGTs is their low power dissipation (*P*), which is expressed by Equation (5)

(5) $$P=V_{\mathrm{DSAT}}\times I_{\mathrm{DSAT}}$$

Furthermore, the extremely flat and stable saturation *I*_DS_ in output characteristics means enhanced output impedance, leading to large SGT intrinsic gains, as expressed by Equation (6)

(6) $$A_{v}=g_{m}\times \gamma =\frac{\partial I_{\mathrm{DS}}}{\partial V_{\mathrm{GS}}}*\frac{\partial V_{\mathrm{DS}}}{\partial I_{\mathrm{DS}}}$$

where *g*_m_ is transconductance, and *γ* is output resistance, making SGTs exceptional candidates for applications in analogue circuits, sensors, and large‐area displays. As a consequence of the power dissipation and intrinsic gains, the saturation voltage and current are major SGT performance metrics, in addition to the transconductance and output resistance, which are extracted from the transfer and output characteristic curves, respectively, are also two major performance parameters. However, note that the major limitation of SGTs is that *I*_DS_ and the transconductance are usually lower than in TFTs. These issues can be minimized by device downscaling, considering the high immunity of SGTs to short‐channel effects. **Table** **1** provides as detailed performance comparison between SGTs and TFTs.

**Table 1:** Performance comparison between traditional TFTs and SGTs

Parameter	TFTs	SGTs
*V*_DSAT_	High *V*_GS_ dependence	Low *V*_GS_ weak dependence
*I*_DSAT_	High	Low
Power consumption	High	Low
Gain	Low	High
*I*_off_	Moderate	Low
Transconductance	High	Low
Resistance	Low	High
Immunity to short channel effects	Poor	Excellent

## 3 Recent Progress in High‐Performance SGTs

As discussed in Section 2, the unique SGT operation mechanism versus that of conventional TFTs accounts for their superior characteristics and has attracted intensive research efforts in recent years. Since the first demonstration of SGT in 2003,^[^ ^33^ ^]^ significant progress has been made in high‐performance SGTs with various semiconductors, including silicon, metal oxides, organic materials, and 2D materials.

### 3.1 Silicon‐Based SGTs

**Figure 3:**
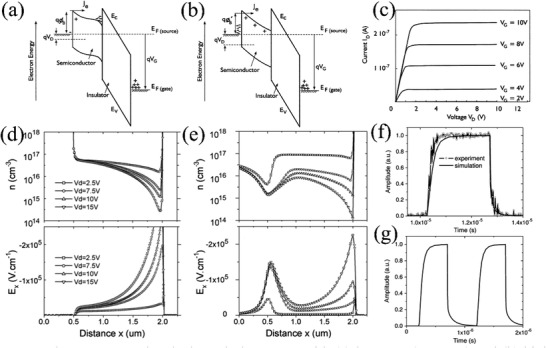
Electron energy bands through the source with a) low *V*_DS_ (<*V*_DSAT_) and b) high *V*_DS_ (>*V*_DSAT_); note in both cases, high *V*_GS_ is applied. c) Output characteristics of the SGT. a–c) Reproduced with permission.^[^ ^33^ ^]^ Copyright 2003, IEEE. Electron concentration (*n*) and the electric field (*E_x_*) for d) TFT and e) SGT along the semiconductor–insulator interface. f) Experimental and simulated transient *I*_DS_ responses at source barrier of 0.49 eV with 100 nm a‐Si:H and 300 nm SiN. g) Simulation for transient responses at source barrier of 0.4 eV with 40 nm a‐Si:H and 120 nm SiN. d–g) Reproduced with permission.^[^ ^58^ ^]^ Copyright 2007, IEEE.

Silicon, the most frequently used semiconductor in modern electronics, was the first channel material adopted in SGTs. a‐Si:H based SGTs were first developed by Shannon et al in 2003, in which a Schottky barrier was created between 100 nm a‐Si:H and a chromium source electrode, which controls the current flow and prevents short‐channel effects.^[^ ^33^ ^]^ Here, 300 nm silicon nitride was used as the dielectric layer on a glass substrate. This work pioneered the SGT concept and emphasized its advantages including a low *V*_DSAT_ and high gains. The energy band bending illustration under the source region is presented in **Figure** **3**a,b when a large positive *V*_GS_ is applied. A low *V*_DS_ (<*V*_DSAT_) reversely biases the Schottky barrier and leads to the expansion of the depleted region (Figure 3a). As *V*_DS_ is increased (≥*V*_DSAT_), the depletion envelope below the source further extends to the semiconductor‐dielectric interface (Figure 3b). Consequently, when *V*_DS_ is larger than *V*_DSAT_, the source and gate electrodes are separated by two tandem dielectrics layers. Therefore, a linear relation exists between the variation of *V*_GS_ and that of the electric field through the source barrier. Thus, *I*_DS_ is dominated by the reverse current from thermionic‐field emission which depends on the electric field. From this model, the SGT impedance is dominated by the variation of electric field at the source barrier, whereas in conventional a‐Si TFTs, it is controlled by channel conductance. As calculated from Figure 3c, this device exhibits a high output impedance along with much lower *V*_DSAT_ (<2 V) and a larger gain (≈85).

In addition to the high gain and low *V*_DSAT_, good tolerance to short‐channel effects allows SGTs to perform well even when fabricated with disordered and poor‐quality semiconductors. This is extremely important, since to achieve successful fabrication on flexible substrates, the semiconductor films are usually deposited at low temperatures affording amorphous/‐microcrystalline films, which have significant defects and trap states.^[^ ^59^, ^60^ ^]^ While conventional TFTs based on these disordered semiconductors have limitations, including electrical/ambient instability and low circuit speeds, the SGT architecture mitigates these issues by employing high internal fields (small channel dimensions) and low carrier concentrations.^[^ ^61^ ^]^

Therefore, by adopting an SGT structure, Shannon et al. further achieved high‐performance devices with good frequency response and excellent stability with a poorly performing semiconductor such as a‐Si:H. Note, a‐Si:H is intrinsically unstable with a high defect concentration of which lowers the carrier mobility.^[^ ^58^ ^]^ For device fabrication, a chromium gate metal electrode was deposited on glass substrates, followed by deposition of silicon nitride and a‐Si:H using plasma‐enhanced chemical vapor deposition (PECVD) at 250 °C. Chromium source and drain Schottky contacts were next deposited and defined after phosphorous donor implantation, which controls the effective Schottky barrier height. The authors carried out simulations of SGT and conventional TFT performance, demonstrating that the SGT carrier concentration decreases toward the edge of the source due to the formation of a depletion envelope (Figure 3d,e), which leads to a stable *I*_DS_ (less than 2% variation) after applying constant *V*_GS_ and *V*_DS_ at 30 °C for 24 h. The higher electric field at the source end (six times greater than that in an equivalent TFT, Figure 3d,e) also contribute to reducing the transit time and enhancing the frequency response. As shown in Figure 3f, the simulated and experimental data on transient *I*_DS_ show high frequency response (1 MHz). Furthermore, as indicated in Figure 3g, the use of a lower Schottky source barrier, or thinner dielectric and semiconductor layers can further increase the cutoff frequency (*f*_T_) to 5 MHz. Moreover, *f*_T_ can be increased by decreasing the source barrier or increasing the gate voltage until it becomes transit time‐limited at ≈20 MHz. In comparison, an equivalent TFT becomes transit time limited at ≈15 MHz because of lower internal fields. The simulated results show a high *f*_T_ of 25 MHz at 10 V bias drain voltage and high electric field of >3 × 10^5 ^V cm^−1^ with more than 100 MHz transit time limit. These results indicate that high‐performance SGTs can be fabricated even with disordered and poor‐quality semiconductor materials.

**Figure 4:**
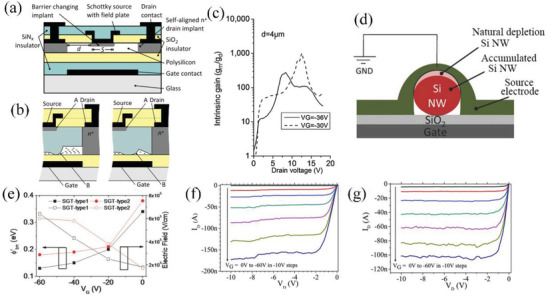
a) Cross‐section illustration of a self‐aligned SGT device structure. b) Schematic of the two pinch off regimes and c) intrinsic gain characteristics of a self‐aligned SGT. a–c) Reproduced with permission.^[^ ^62^ ^]^ Copyright 2010, IEEE. d) Schematic of SGT based on Si NWs. e) Extracted barrier heights and electric field inside the depletion region as a function of *V*_GS_ for SGTs (SGT‐type1: Ni as S/D contacts, SGT‐type2: W as S/D contacts). Output characteristics of SGTs with f) Ni S/D contacts and g) W S/D contacts. d–g) Reproduced with permission.^[^ ^55^ ^]^ Copyright 2016, WILEY‐VCH.

In 2010, by utilizing self‐aligned polysilicon based SGT architecture, an intrinsic gain of 1000 was obtained by Sporea et al.^[^ ^62^ ^]^ As shown in **Figure** **4**a, 200 nm SiN*_x_* and 200 nm SiO_2_ were deposited as dielectric layer using PECVD, followed by 40 nm of a‐Si:H. The amorphous silicon was dehydrogenated by baking at 450 °C to form polysilicon, and BF_2_ or P implants were adopted to dope the polysilicon. A high *P* concentration was used to form the drain‐contact regions aligned with the gate. Chromium and Al/Ti metal layers were then deposited and patterned to form a Schottky‐source and field‐plate structure. Finally, the device was passivated using a 0.6 µm thick Si_3_N_4_ layer. Using this architecture, the authors expected to suppress the current collapse phenomena (so‐called kink effect) to achieve a very large output impedance. Note, in this device, another pinch off region is observed during operation: further increasing *V*_DS_ to *V*_sat2_ can induce a new pinch‐off region close to the drain contact, as shown in the right panel of Figure 4b. These pinch‐off regions lead versus ohmic contacts on the transistor to the generation of two peaks in the intrinsic gain curve (Figure 4c) with the highest gain achieved when both the source and drain are pinched‐off. Such properties enable SGTs with high gains or high output impedances for analog applications through proper design and optimization.

In order to address the inferior mechanical flexibility of bulk silicon, silicon nanowires (Si NW) were introduced in SGTs due to their low cost, high yield, mechanical flexibility and adjustable semiconductor performance.^[^ ^63^, ^64^ ^]^ Shkunov et al.^[^ ^55^ ^]^ fabricated SGTs with silicon nanowire arrays by spray‐coating suspensions of NWs onto clean Si/SiO_2_ substrates (230 nm SiO_2_ dielectrics). The typical NW length and diameter were around 5–40 µm and 20–30 nm, respectively (Figure 4d). Au, Ni, and W source/drain electrodes were used to investigate the influence of the Schottky versus ohmic contacts on the transistor characteristics. The effective barrier height of Au, Ni, and W contacts is 0.1, 0.34, and 0.38 eV at *V*_GS_ = 0 V. With the aid of 2D numerical simulations, the authors found that the source barrier depends on, and can be reduced by *V*_GS_ (Figure 4e). Source barrier lowering induced by *V*_GS_ was found to be ≈3 meV V^−1^ for the Ni and W contacts, accessed by activation energy measurements. While introducing a high source contact barrier (Ni and W electrodes) contributes to earlier pinch‐off and sudden current saturation at low *V*_DS_ (≈1 V), the device exhibits typical SGT characteristics (Figure 4f,g). The extracted Δ*V*_DSAT_/Δ*V*_GS_ ratios (0.03 with Ni electrodes and 0.02 with W electrodes) are much smaller than 0.73 in a TFT, demonstrating that *V*_DSAT_ of these SGTs is stable under different *V*_GS_. Such devices also operate at low currents (<1 µA) with a high on/off current ratios (10^4^–10^5^) which are suitable for low power applications, such as self‐powered autonomous sensors, wearable electronics, and analog circuits.

### 3.2 Metal Oxide Semiconductor‐Based SGTs

In addition to Si, metal oxide semiconductors have been investigated for high‐performance SGTs. Among them, zinc oxide (ZnO) was the first oxide semiconductor used in SGTs because of its high electron mobility, wide bandgap, good stability, facile low‐temperature synthesis, and high breakdown fields.^[^ ^65^ ^]^ In 2012, Barlage et al. employed ZnO thin films as the active layer for SGT deposited by pulsed laser deposition.^[^ ^44^ ^]^ Gold and aluminum electrodes were utilized as the source Schottky barrier and ohmic drain contacts, respectively. The Schottky barrier height is calculated ranging from 0.6 to 0.8 eV.^[^ ^66^ ^]^ Such devices exhibit high field effect mobilities (≈0.1 cm^2^ V^−1^ s^−1^), high *I*_on_/*I*_off_ (≈10^5^), and low *V*_DSAT_ (≈6 V). By analyzing the transconductance– and capacitance–voltage characteristics, it was found that the carrier injection mechanism across a Schottky barrier converts from thermionic emission to tunneling at a gate bias of approximately 8 V. Such SGT architectures provide a simple approach for engineering enhancement mode ZnO‐based transistors, which are attractive for low‐cost power inverters and radio‐frequency identification (RFID) tags, and transparent electronics applications.

**Figure 5:**
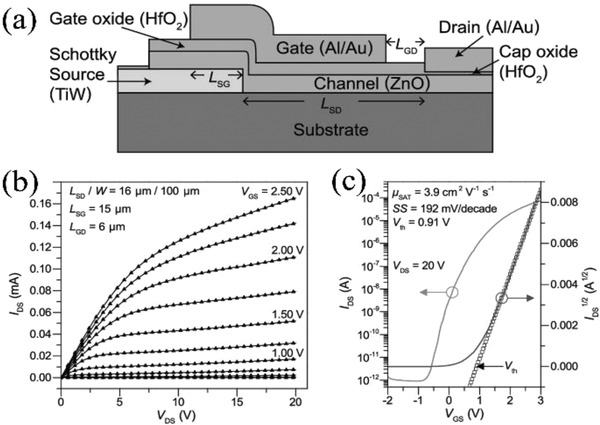
a) Configuration, b) output characteristics, and c) transfer characteristics of a ZnO SGT. Reproduced with permission.^[^ ^67^ ^]^ Copyright 2013, American Institute of Physics.

In 2013, the same group fabricated ZnO SGTs by depositing the ZnO layer via atomic layer deposition.^[^ ^67^ ^]^ The device reported in **Figure** **5**a utilizes TiW (12 nm thick) and Al/Au metals used as the Schottky source electrode and ohmic drain electrode, respectively. The devices exhibit excellent saturation characteristics, and the output conductance (*g*_ds_) increases from 3.0 nS to 3.6 µS with *V*_GS_ increasing from 0.25 to 2.5 V (Figure 5b). A high drain current per unit width (1.6 mA mm^−1^) is obtained at *V*_DS_ = 20 V and *V*_GS_ = 2.5 V. A rectifying behavior at the source junction makes the major contribution to g_ds_. Such devices exhibit near‐zero *V*_T_ (0.91 V), a high electron mobility (3.9 cm^2^ V^−1^ s^−1^), low subthreshold swing (192 mV per decade) and a high *I*_on_/*I*_off_ (≈7 × 10^7^), as shown in Figure 5c. Moreover, the breakdown voltage (*V*_BD_), higher than >20 V, is achieved by reduced peak electric field at the drain edge due to the unique device architecture (buried source and top gate/drain device structure).

**Figure 6:**
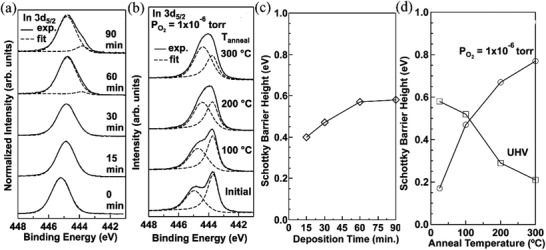
Normalized In 3d_5/2_ core‐level IGZO XPS data a) after different Pt deposition durations and b) after different annealing temperatures at *P*_O2_ = 1 × 10^−6^ Torr. Schottky barrier height versus c) Pt deposition durations and d) annealing temperature in different ambient. Reproduced with permission.^[^ ^69^ ^]^ Copyright 2018, American Chemical Society.

Another important metal oxide semiconductor, indium gallium zinc oxide (IGZO), has also attracted much research interest for large area and low power consumption electronics due to the amorphous nature, superior mobility, low temperature processability, and low *I*_off_.^[^ ^25^, ^59^, ^68^ ^]^ Herman et al. investigated the effect of interfacial chemistry on the Schottky barrier heights between a Pt source electrode and amorphous IGZO through background ambient O_2_ pressure control and subsequent thermal processing.^[^ ^69^ ^]^ Thus, 50 nm a‐IGZO films were deposited on TiN/Ti/SiO_2_/Si substrates by radio frequency sputter deposition using an InGaZn_0.5_O_3.5_ sputtering target, and the films were then heated to 300°C for 10 min in the ultrahigh vacuum chamber at *P*_O2_ = 1.0 × 10^−6^ Torr to completely reoxidize the a‐IGZO surface. Afterward, Pt was sequentially deposited by e‐beam evaporation. The authors found that In^3+^ at the IGZO surface is reduced to In^0^ during the deposition of Pt in ultrahigh vacuum (6.0 × 10^−9^ Torr) (**Figure** **6**a).

Further annealing in an O_2_ ambient re‐oxidizes In^0^ at the interface and leads to an increased Schottky barrier height (Figure 6b). The Schottky barrier height also increases and can reach 0.58 eV as Pt deposition time increases (Figure 6c). Annealing devices after Pt deposition in different ambient also contributes to variation in barrier height, which decreases from 0.58 to 0.21 eV when annealing in ultrahigh vacuum while increases from 0.17 to 0.77 eV when annealing to 300 °C in P_O2_ = 1 × 10^−6^ Torr (Figure 6d). The reduction of the a‐IGZO interface indicates the formation of Pt—O bonding,^[^ ^70^ ^]^ and increases the effective Schottky barrier height. Therefore, thermal processing and O_2_ partial pressure control can tune the indium reduction and modulate Schottky barrier heights from 0.17 to 0.77 eV, which paves the way for desirable IGZO‐based SGT fabrication.

**Figure 7:**
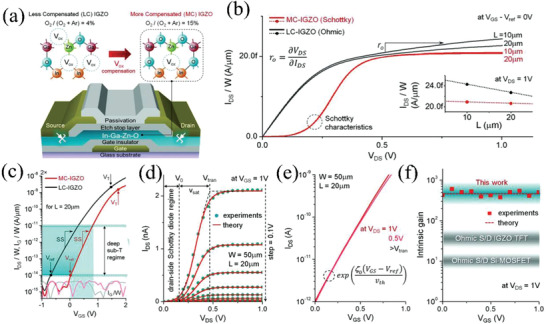
a) Schematic cross‐section of an IGZO SB‐TFTs. b) Measured *I*_DS_/*W* versus *V*_DS_ and c) Measured input characteristics of MC‐IGZO and LC‐IGZO based transistors. d) Measured and theoretically simulated output characteristics of SB‐TFTs in the deep subthreshold regime. e) Transfer characteristics of SB‐TFTs at *V*_DS_ = 0.5 and 1 V. f) Intrinsic gain versus gate bias. Reproduced with permission.^[^ ^71^ ^]^ Copyright 2016, American Association for the Advancement of Science.

Similarly, Nathan et al. achieved high‐performance subthreshold Schottky‐barrier thin‐film transistors (SB‐TFTs) by controlling annealing temperature and O_2_ partial pressure during IGZO sputtering (P_ox_).^[^ ^71^ ^]^ Such devices could not create depletion envelope due to the lack of gate‐source overlap, which is different with SGTs. High oxygen partial pressure was utilized to compensate for oxygen vacancies acting as electron donors, which leads to formation of desirable Schottky contact. As shown in **Figure** **7**a, the Mo gate electrode deposited onto glass substrates via radio‐frequency (RF) sputtering, and then SiO*_x_* and SiN*_x_* layers were grown as gate dielectrics using PECVD. A 50 nm‐thick IGZO film with target composition of In_2_O_3_:Ga_2_O_3_:ZnO = 1:1:2 was deposited using RF‐sputtering an O_2_ partial pressure versus Ar (i.e., 15%: 4%). Subsequently, SiO*_x_* was used as etch‐stop layer followed by 150 nm‐thick Mo deposition as source/drain electrodes. Indeed, the more compensated (MC) IGZO TFTs at *P*_ox_ = 15% exhibit Schottky characteristics while less compensated (LC) IGZO TFTs at *P*_ox_ = 4% exhibit ohmic behavior. The output curve of the MC‐ TFTs is much flatter than that of LC‐TFTs, yielding a much lower output conductance (Figure 7b). Furthermore, the MC‐ TFTs could operate in a deep subthreshold regime and has small subthreshold slope (SS) of 0.28 V per decade compared with 0.34 V per decade in the ohmic device, which indicates a higher transconductance (*g*_m_) and reduced defects with higher *P*_ox_. The result is an ultralow supply power < 1 nW is obtained in view of the low operating current ranging from pA to nA and small voltage < 1 V (Figure 7c,d). Such devices exhibit almost identical transfer characteristics at *V*_DS_ = 0.5 and 1 V, implying that the drain current reaches saturation when *V*_DS_ > 0.48 V (Figure 7e). The intrinsic gain is calculated to be ≈450 and is an order of magnitude higher than that of ohmic IGZO TFTs (Figure 7f). Such devices are electrically stable for 10 000 s under voltage bias due to low current and low driving voltage. Moreover, it was shown that the source Schottky contact quality improvement can decrease the ideality factor (*n*), hence increase the output resistance and intrinsic gain.

**Figure 8:**
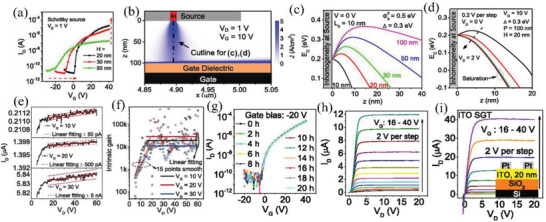
a) Transfer characteristics of IGZO SGTs with different IGZO thickness. b) Current density distribution in the SGT with 100 nm thick IGZO (IH represents inhomogeneity). c) Profiles of the conduction band minimum beneath the inhomogeneity with different channel thicknesses at zero bias. d) Profiles of the conduction band minimum beneath the center of the SGT inhomogeneity with 20 nm thick IGZO versus drain bias. e) Output curves with 20 nm thick IGZO at different gate bias, and f) corresponding intrinsic gains. g) Transfer curves under negative bias illumination temperature stress for 20 h. h) Output curves of short‐channel SGTs with channel length of 360 nm. i) Output curves of SGT with ITO channel. Reproduced with permission.^[^ ^72^ ^]^ Copyright 2019, National Academy of Sciences.

Based on the aforementioned research achievements, IGZO SGTs with extremely high intrinsic gain were obtained by Song et al.^[^ ^72^ ^]^ Thus, 10–100 nm thick IGZO films were grown on SiO_2_‐Si wafers with 100 nm thick SiO_2_ using RF sputtering and an IGZO target with an atomic ratio of 1:1:2 (In_2_O_3_:Ga_2_O_3_:ZnO), followed by Pt deposition as source/drain contacts via RF sputtering. As the IGZO thickness decreases, the turn‐on voltage (*V*_ON_) shifts toward positive *V*_GS_ direction, reflecting easier depletion for a thinner active layer (**Figure** **8**a). A barrier inhomogeneity (IH, a region of lower barrier height) was introduced during the SGT simulation and indicated that the current is dominated by the contribution from the lower barrier inhomogeneity (Figure 8b). Thinner semiconductor layers also contribute to a flatter saturation current, which was further explained by device simulations with barrier inhomogeneity. As shown in Figure 8c,d, a saddle point (the point in the conduction band minimum where the derivative is zero) exists beneath the inhomogeneity for a thicker semiconductor. The authors pointed that with the semiconductor thickness decreasing, thus the electric field increasing, the saddle point height could be effectively lowered or even removed. After saturation, the reduction of the saddle point results in flattering the saturation current (Figure 8e), achieving an extremely high intrinsic gain. Therefore, intrinsic SGT gains with a 20 nm thick IGZO layer were calculated to be 19 000, 29 000, and 11 000 at *V*_GS_ = 10, 20, and 30 V, respectively (Figure 8f). For the 20 nm thick IGZO SGT, immunity to negative bias illumination temperature stress was also observed, exhibiting enormous potential for display technologies (Figure 8g). Furthermore, transistors maintaining a flat saturation current with a channel length of only 360 nm were also realized, indicating the excellent SGT immunity to short‐channel effects (Figure 8h). More interesting, the authors demonstrated that semimetal‐like oxide ITO can also be utilized as the SGT active layer resulting in devices with respectable performance, especially the flat saturation current (Figure 8i), which broadens the choices of channel materials.

**Figure 9:**
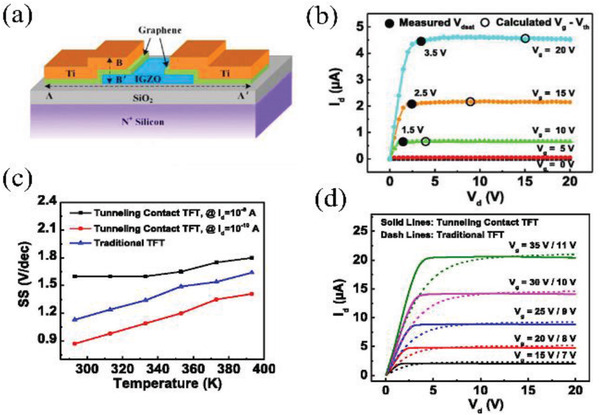
a) Schematic structure and b) output characteristics of tunneling contact IGZO SGTs, where *V*_DSAT_ are significantly smaller than those in the traditional transistors of *V*_GS_–*V*_T_. c) SGT and TFT subthreshold swing dependence on temperature. d) Output characteristics of the tunneling contact SGTs and traditional TFT with similar drain current. Reproduced with permission.^[^^]^ Copyright 2017, American Institute of Physics.

As discussed above, diffusion of metal or oxygen at the source contact‐semiconductor interface influence the Schottky contact barrier height, which may affect long term stability. Zhang et al. demonstrated tunneling contact SGTs with a graphene interlayer between amorphous IGZO and Ti electrodes,^[^ ^73^ ^]^ since graphene is capable of modifying the contact properties between the semiconductor and the electrode.^[^ ^74^ ^]^ graphene monolayer was grown on copper by chemical vapor deposition and transferred to the IGZO film surface to realize Schottky contacts with Ti electrodes (**Figure** **9**a). The graphene monolayer prevents elemental diffusion and forms an effective potential barrier of 0.2 eV. Hence, a Schottky contact is formed, leading to a significant difference in *V*_DSAT_ versus traditional TFTs (Figure 9b). The tunneling contact also exhibited a new transport mechanism. Specifically, while traditional TFTs with ohmic contacts show a “kT” behavior, indicating the dominance of thermionic emission (Figure 9c), in these graphene‐tunnel SGTs the subthreshold swing temperature dependence changes from strong to weak as the drain current is increased. This result suggests that the electron transport mechanism transitions from thermionic emission dominated to quantum tunneling dominated. Moreover, as shown in Figure 9d, with a similar drain current, obviously lower *V*_DSAT_s are observed in the graphene‐tunnel SGTs compared to those in traditional TFTs, which would lower power consumption.

**Figure 10:**
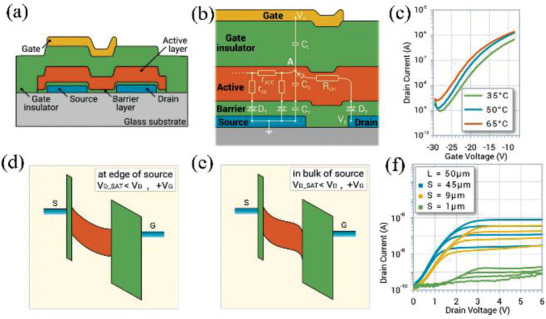
a) Structure of the tunnel‐contact SGTs and b) equivalent circuit model of their function. c) Transfer characteristics under different temperatures. Band diagram d) at edge of source and e) in bulk of source. f) Output curves with different source length (*S*). Reproduced with permission.^[^ ^57^ ^]^ Copyright 2019, WILEY‐VCH.

Sporea et al. also reported tunnel‐contact SGTs but with a 3 nm‐thick Al_2_O_3_ film between the IGZO film and Ni electrodes.^[^ ^57^ ^]^ As shown in **Figure** **10**a, such SGTs were constructed with top‐gate, bottom‐contact configurations having a source‐gate overlap, *S*, and a source–drain gap, *L*. An equivalent circuit model to illustrate the device operation is shown in Figure 10b. Such devices exhibit more than a five‐fold increase in *I*_DS_ over a 30 K change, which indicates that barrier‐layer tunneling is not the principal current control mechanism and a more complex operation mechanism must apply (Figure 10c). Thus, under a higher *V*_DS_ (>*V*_DSAT_), the capacitance of the depleted semiconductor is far less than that of the tunneling layer (Al_2_O_3_). The current is therefore dominated by the emission of carriers injected from the source end into the depletion region, which is in high‐field mode (Figure 10d). As for the source region overlapped by the gate, the carriers are accumulated at the insulator interface. Thus, the current is dominated by the current through the source bulk, which is identified as in low‐field mode (Figure 10e). To explore this behavior further, the authors fabricated devices with different source lengths (*S* = 1, 9, and 45 µm), and the results show that the *S* value affects the saturation current and thus the device operation mode. For a small *S* value (1 µm), the devices operate in the high electric field mode and a small potential across the Al_2_O_3_barrier layer yields a low current. In contrast, for devices with a large *S* (9 and 45 µm), a low electric field mode with much higher current dominates and affords a small dependence of *I*_DS_ on S (Figure 10f). In addition, the tunnel‐contact SGTs show flat output characteristics with a low saturation voltage (≈3 V) and a small dependence on *V*_GS_ (≈0.12). The authors concluded that these devices could be optimized further to increase the gain by introducing a field plate in source contact area and reducing the semiconductor thickness.

### 3.3 Organic SGTs

Organic semiconductors are promising for next‐generation electronics due to their low processing temperatures, light weight, mechanical flexibility, and, with small molecules, well‐defined molecular structures.^[^ ^75^, ^76^ ^]^ Since the 1970s, structural variations of several organic semiconductor families has enabled impressive advances in important semiconducting performance properties including mechanical/impact resistance, light absorption/emission, and magnetic/charge transport.^[^ ^77^, ^78^, ^79^, ^80^ ^]^ Moreover, organic semiconductors have been effectively integrated into opto‐electronic devices such as light‐emitting diodes, different types of solar cells, thin‐film transistors/ RFID tags, and diverse sensors.^[^ ^81^, ^82^, ^83^, ^84^ ^]^

**Figure 11:**
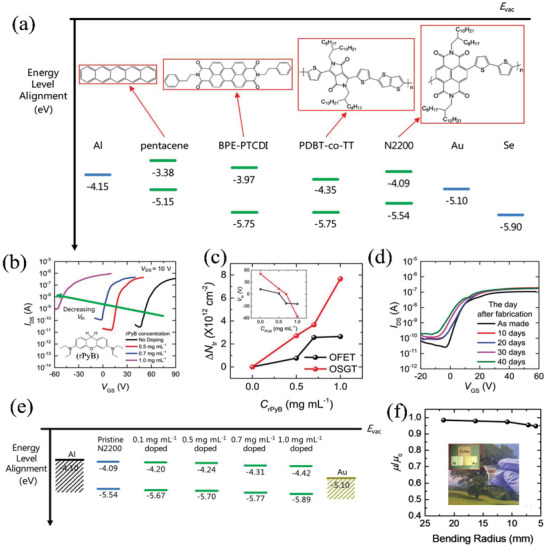
a) Energy levels of organic semiconductors and metals discussed here. b) Transfer curves of rPyB‐doped N2200 OSGTs; insert: chemical structure of rPyB. c) Filled trap density versus rPyB dopant concentration. d) Transfer curves of 0.7 mg mL^−1^ rPyB ‐doped N2200 OSGTs in air for up to 40 d. e) Energy levels of rPyB‐doped N2200 films. f) Electron mobility of flexible BPE‐PTCDI OSGTs after bending. Reproduced with permission.^[^ ^87^ ^]^ Copyright 2019, WILEY‐VCH.

Organic semiconductors also show great potential in SGTs, especially for wearable electronics, due to their excellent mechanical flexibility.^[^ ^85^, ^86^ ^]^ Since the first demonstration of an organic SGT (OSGT) in 2013,^[^ ^54^ ^]^ Oh et al.^[^ ^87^ ^]^ systematacially reported OSGTs with a bottom‐gate top‐contact configuration based on four representative organic semiconductors including the small molecules pentacene and *N*,*N*′‐bis(2‐phenylethyl)‐perylene‐3,4:9,10‐tetracarboxylic diimide (BPE‐PTCDI), as well as the polymers poly{2,2′‐[(2,5‐bis(2‐octyldodecyl)‐3,6‐dioxo‐2,3,5,6‐tetrahydropyrrolo[3,4‐c]pyr‐role‐1,4‐diyl)]dithiophene‐5,5′‐diyl‐*alt*‐thieno[3,2‐b]thiophen‐2,5‐diyl} (PDBT‐*co*‐TT) and poly{[*N*,*N*′‐bis(2‐octyldodecyl)‐naphthalene‐1,4,5,8‐bis(dicarboximide)‐2,6‐diyl]‐*alt*‐5,5′‐(2,2′‐bithiophene)} (N2200). The chemical structures and energy levels of these organic materials are shown in **Figure** **11**a. Pentacene and BPE‐PTCDI were thermally deposited under high vacuum onto Si/SiO_2_ substrates (300 nm SiO_2_), while PDBT‐*co*‐TT and N2200 were spin‐coated in an N_2_ environment. Three electrode types were grown for OSGTs by thermal evaporator using shadow masks: Al as the drain electrode and Au (or Se) as the source electrode for n‐type semiconductors whereas Al as the source electrode and Au (or Se) as the drain electrode for p‐type semiconductors. SGTs based on the above four organic semiconductors exhibit higher *I*_on_/*I*_off_ (10^6^–10^7^) and lower *V*_DSAT_ (<10 V) compared with the corresponding organic thin‐film transistors (OTFTs). However, the |*V*_T_| was increased.

To decrease the *V*_T_ of the N2200 devices, reduced Pyronin B (rPyB; Figure 11b) was used as an n‐type dopant, which was spin‐coated on the surface of N2200 before source/drain electrode deposition. The transfer curves in Figure 11b show that as the concentration of rPyB increases, *V*_T_ shifts negatively. Thus, free charge carriers created by the n‐doping process fill deep electron traps and optimize *V*_T_ for a rPyB concentration of 0.7 mg mL^−1^ (Figure 11c). Moreover, air instability is a major issue for n‐type organic semiconductors due to their relatively shallow lowest unoccupied molecular orbital (LUMO) levels, but here it was found that N2200 based OSGTs coated with 0.7 mg mL^−1^ rPyB retain >90% of the original electron mobility (≈0.033 cm^2^ V^−1^ s^−1^) after 49 d in air (Figure 11d). Improved air stability by rPyB doping was ascribed to: 1) Energetically, rPyB‐doped N2200 has a lower LUMO energy (−4.42 eV for 1.0 mg mL^−1^ rPyB doped N2200) versus −4.09 eV of pristine N2200 (Figure 11e); 2) Kinetically, the rPyB layer on top of N2200 suppresses diffusion of ambient oxidants. Finally, 7 × 7 BPE‐PTCDI flexible OSGT arrays were successfully fabricated on Parylene‐C substrates and exhibit a mobility retention of ≈95% after bending at a radius of 5.8 mm (Figure 11f).

**Figure 12:**
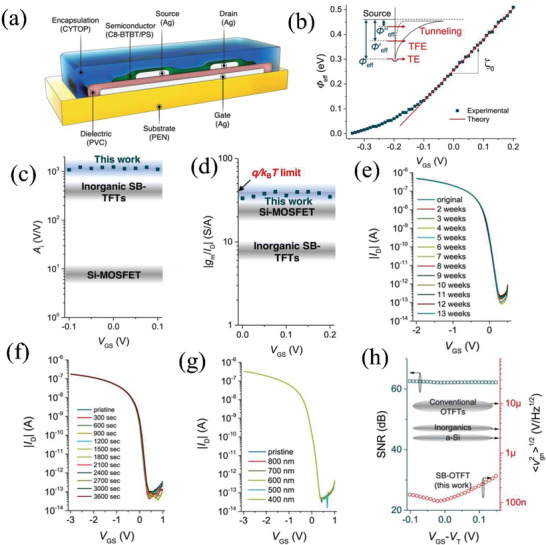
a) Schottky barrier SB‐OTFT structure. b) Effective Schottky barrier heights versus gate voltage. c) Measured intrinsic gain versus gate voltage comparison for a SB‐OTFT, inorganic SB‐TFTs, and Si‐MOSFETs. d) Experimental transconductance efficiency (*g*_m_/*I*_D_). e) Transfer curves of SB‐OTFT under ambient over 13 weeks of aging. f) Transfer curves under negative bias stress (*V*_GS_ = *V*_DS_ = −3 V) for up to 3600 s. g) Transfer curves under light exposure. h) Signal‐to‐noise ratio and input‐referred voltage noise comparison for SB‐OTFT, inorganic SB‐TFTs, and Si‐MOSFETs. Reproduced with permission.^[^ ^88^ ^]^ Copyright 2019, American Association for the Advancement of Science.

Nathan et al. fabricated subthreshold Schottky barrier (SB) OTFTs with high gain and ultralow power consumption via inkjet‐printed circuitry.^[^ ^88^ ^]^ Here the bottom‐gate bottom‐contact SB‐OTFT used silver as the source/drain/gate electrodes, polyvinyl cinnamate (PVC) as the gate dielectric, 2,7‐dioctyl[1]benzothieno[3,2‐b][1]benzothiophene (C8‐BTBT) blended with polystyrene (PS) as the low HOMO level p‐type semiconductor layer with large phase‐separated crystals (>50 mm), and CYTOP for encapsulation (**Figure** **12**a). All materials were formulated for the inkjet printing. A smooth interface with an RMS roughness ≈0.21 nm is formed between the semiconductor and dielectric, which effectively reduces carrier trapping and scattering effects. The authors did not report the field‐effect mobility, however, these exhibit near‐zero *V*_T_ (≈−0.01 V), a high *I*_on_/*I*_off_ (>10^7^), low operation voltage (<1 V), and an ideal subthreshold swing of ≈60.2 mV per decade, which benefit from the large semiconductor crystal size that reduces the total number of grain boundaries and stacking faults, lowering the defect density. Operation in the subthreshold regime enables a good initial Schottky barrier (≈0.51 eV) and channel current is mainly dominated by thermionic emission rather than thermionic field emission or tunneling effects (Figure 12b). The low defect density and high Schottky barrier in the subthreshold regime lead to an ultrasteep subthreshold slope and a high transconductance, affording a gain of ≈1100 and signal amplification efficiency (*g*_m_/*I*_DS_) of ≈38.2 S A^−1^, the latter approaching the theoretical value of 38.7 S A^−1^ (Figure 12c,d). In addition, as shown in Figure 12e–g, high stability in ambient and almost complete immunity to negative bias illumination stress are obtained. Finally, a high signal‐to‐noise ratio of 63 dB was also achieved in the subthreshold regime (Figure 12h), making the devices suitable for analog applications.

### 3.4 2D Material‐Based SGTs

**Figure 13:**
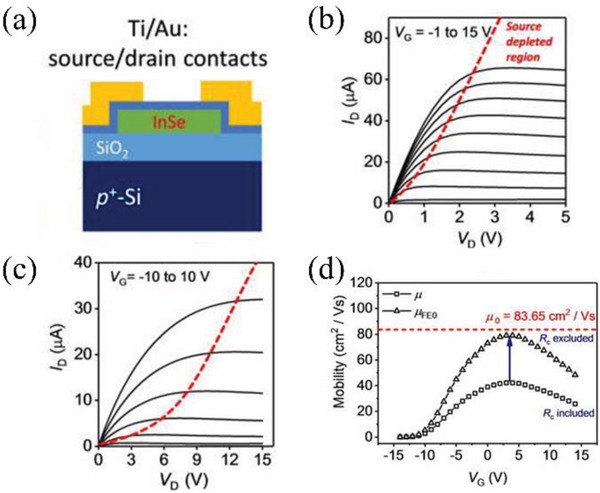
a) Structure of an InSe SGT with Ti/Au source/drain contacts. Output characteristics of b) an InSe SGT and c) an InSe TFT with ohmic contacts. d) InSe SGT mobility versus *V*_G_ characteristics. Reproduced with permission.^[^ ^89^ ^]^ Copyright 2019, American Institute of Physics.

SGTs have also incorporated 2‐D semiconductors owing to their superior electronic properties.^[^ ^41^, ^48^ ^]^ Song et al. reported SGTs based on a InSe nanosheet semiconductor coated with a HfO_2_ layer before deposition of the source/drain electrodes.^[^ ^89^ ^]^ As shown in Figure 10a, a heavily p‐doped Si substrate with 100 nm SiO_2_ dielectric was used as the bottom‐gate electrode. Then InSe flakes were transferred from a Bridgman‐grown InSe crystal onto the substrate, followed by the deposition of a 0.9 nm thick HfO_2_ film using atomic‐layer deposition. Ti/Au (20 nm/50 nm) source/drain electrodes were then deposited by electron‐beam evaporation with channel length *L* = 10 µm and width *W* = 10 µm. A 0.9 nm thick HfO_2_ film was used to encapsulate 50 nm thick InSe nanosheet and can lower the Schottky contact resistance (6.63 kΩ) due to a stronger interface dipole effect, leading to more effective gate modulation. These SGTs exhibit a much lower *V*_DSAT_ (<2 V) versus that (>10 V) in the corresponding ohmic‐contact TFT (**Figure** **13**b,c) fabricated p‐doped Si wafer (back gate) with a 300 nm thick thermally grown SiO_2_ dielectric layer. However, for the SGT, the mobility cannot be calculated from the standard linear transfer model due to the considerable contact resistance (*R*_c_). Therefore, the Y‐function method $[Y=\frac{I_{\mathrm{DS}}}{{g_{m}}^{1/2}}={\left(\frac{W}{L}C_{\mathrm{ox}}\mu V_{D}\right)}^{\frac{1}{2}}\;\left(V_{\mathrm{GS}}-V_{T}\right)$] was introduced to determine resistance value and intrinsic field‐effect mobility, where *R*_c_ *W* (200 kΩ µm) of the SGT is higher than that in an ohmic‐contact TFT (≈44 kΩ µm). The extracted intrinsic field‐effect mobility is 83.7 cm^2^ V^−1^ s^−1^ from the Y‐function method (Figure 13d), which agrees well with corrected standard TFT model with the contact and channel resistance. Thus, this study suggested a reliable method to calculate the SGT contact resistance and mobility.

**Figure 14:**
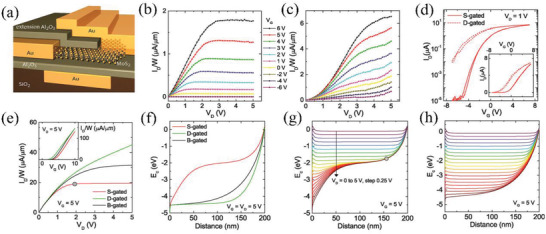
a) Structure of self‐aligned MoS_2_ transistors. Output characteristics of b) source‐gated transistors and drain‐gated MoS_2_ transistors. d) Transfer characteristics of the source‐gated and drain‐gated transistors at *V*_DS_ = 1 V. e) Simulated transfer (inset) and output characteristics of the indicated devices. f) Calculated conduction band edge energy profiles for g) source‐gated transistors and h) traditional back‐gated TFTs versus distance and drain bias. Reproduced with permission.^[^ ^90^ ^]^ Copyright 2018, American Chemical Society.

In addition to a Schottky contact approach, SGTs can also be created by innovative device architecture engineering. Recently, source‐gated transistors were reported using MoS_2_ monolayers by the Hersam group^[^ ^90^ ^]^ where self‐aligned geometries with short channels, dielectric extension, and source contact overlapping the channel are fabricated. Thus, a Au gate contact was defined by e‐beam lithography and thermally evaporated onto 300 nm SiO_2_/Si substrates, followed by ALD growth of a 35 nm Al_2_O_3_ dielectric layer. Then a CVD‐grown MoS_2_ layer was transferred onto these pre‐patterned local‐gates, followed by ALD growth of 30 nm Al_2_O_3_ dielectric extension, and subsequent Au deposition for source/drain electrodes. The source contact overlapping the channel and dielectric extension contribute to field relief, which should minimize short channel effects. As shown in **Figure** **14**a, this geometry enables two different device operation modes depending on the biasing: 1) When the overlapping electrode (top electrode) is grounded and acts as the source electrode, and the electrode beneath the dielectric extension (left electrode) is biased as the drain electrode, it functions as an SGT. However, in a source‐gated arrangement, the carrier density in the channel near the source contact is lower than that in the drain‐gated configuration, and a depletion region emerges near the source electrode. Note that the SGTs have much flatter current saturation than that in drain‐gated transistors (Figure 14b,c). 2) However, when the bias is reversed, this device functions as a drain‐gated transistor. The bias configuration determines whether the overlapping electrode reduces or increases the carrier density in the channel via the field‐effect. When operated as an SGT, the device exhibits a high on/off current ratio of 10^5^, a low saturation voltage (<2 V), a flat saturation current. however, when working as a drain‐gated transistor the device cannot be completely turned off (Figure 14d). To better understand the operation mechanism, the authors investigated in detail by simulating the potential distributions, carrier densities, and resulting charge transport for bias conditions without incorporating quantum effects. Simulated output curves revealed a two‐fold reduced saturation drain voltage and a 7‐fold reduced output conductance for the SGT versus traditional back‐gated TFTs (Figure 14e). Simulated energy band and electric field effects showed a pinch‐off region near the source electrode, which emerged for SGT operation at *V*_DS_ ≥ 2 V and was not observed in the traditional back‐gated TFTs (Figure 14f–h). Output impedance of more than 20 MΩ was also measured, which was higher than in back‐gated MoS_2_ TFTs (≈32 kΩ). Consequently, these promising self‐aligned MoS_2_ devices exhibit near‐ideal saturation characteristics with short‐channels down to 135 nm.

## 4 SGT Applications

**Figure 15:**
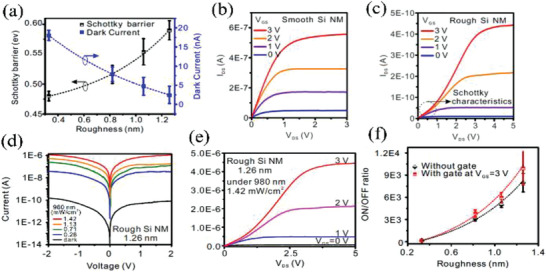
a) Schottky barrier heights and dark current as function of the roughness for Si NM (nanomembrane)‐based diodes. Output characteristics of b) smooth Si NM SB TFTs and c) rough Si NM (1.26 nm) SB TFTs in dark. d) Current−voltage of rough Si NM‐based diode under 980 nm light. e) Output characteristics of rough Si NM SB TFTs under 980 nm light. f) Current on/off ratio for the rough Si NM‐based photodetectors. Reproduced with permission.^[^ ^92^ ^]^ Copyright 2018, American Chemical Society.

SGTs have enormous potential in a number of optoelectronic technologies, and demonstrator applications in photodetectors, inverters, amplifiers, and current‐mode logic circuits. The presence of a Schottky barrier in the SGT architecture depletes the channel of carriers leading to significant dark current suppression. This property is essential for fabricating high performance photodetectors functioning at low driving voltages.^[^ ^91^ ^]^ Thus, Mei and co‐workers reported high‐performance photodetectors based on silicon nanomembrane (Si NM) SB TFTs.^[^ ^92^ ^]^ The authors investigated how varying the Si NM surface roughness affects the Schottky barrier height and optoelectronic response. The Si NM film roughness was varied by depositing a 10 nm thick chromium layer on the 50 nm thick Si NM films followed by etching with a 10% KOH solution for 0–15 s, yielding devices with an RMS roughness ranging from 0.33 to 1.26 nm. The Si NM film surface roughness induces a large number of surface defects, displacing the Fermi energy away from the valence band edges. Thus the barrier height to holes in p‐type Si NMs is increased, which further modifies the dark current. Thus, the dark current falls from 2 to 0.1 nA and the Schottky barrier increases from 0.46 to 0.6 eV as the Si NM roughness is increased from 0.33 to 1.26 nm (**Figure** **15**a). Therefore, compared with the device with a smooth Si NM channel, the device with a rough Si NM (1.26 nm) exhibits a slowly increasing drift at the initial region, caused by the Schottky barrier (Figure 15b,c). For the diodes (no gate electrode), the higher current ratio with/without light illumination reaches ≈7 × 10^3^ for 1.26 nm Si NM, which is attributed to the decreased dark current for the rough contacts (Figure 15d). Compared with operation in the dark, the SB TFTs exhibit 10^4^ times higher current under illumination (Figure 15e). Furthermore, compared with diodes without gate bias, SB TFTs possess enhanced *I*_on_/*I*_off_ ratios (Figure 15f). Compared with a Si nanowire diode (*I*_on_/*I*_off_ = 10^2^), Si NM‐based SB TFTs exhibit a higher *I*_on_/*I*_off_ = 10^4^, making such devices promising for ultrahigh sensitivity optoelectronics. Compared with TFTs, the presence of a Schottky barrier leads to significant dark current suppression and enables SGT‐based photodetectors exhibiting lower current(10^−7^–10^−6^A) and lower driving voltage (<5 V), which implies great potential for lower‐power‐consuming electronics.

**Figure 16:**
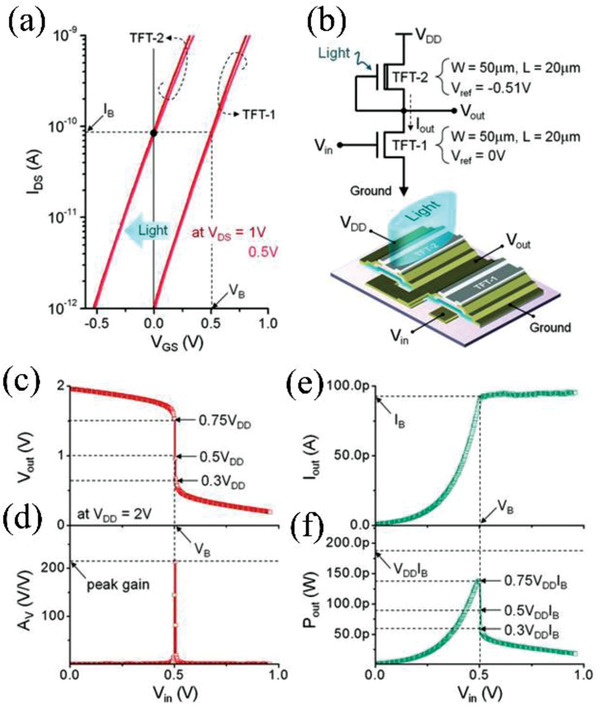
a) Transfer curves of TFT‐1 (IGZO SB‐TFTs) and TFT‐2 (IGZO SB‐TFTs under light stress). b) Common‐source amplifier and its 3D structure based on two SB TFTs. c) Output voltage, d) voltage gain, e) output current, and f) output power consumption of amplifier circuit versus input voltage. Reproduced with permission.^[^ ^71^ ^]^ Copyright 2016, American Association for the Advancement of Science.

Nathan et al. fabricated a common source amplifier based on IGZO SB‐TFTs (**Figure** **16**a,b).^[^ ^71^ ^]^The amplifier consisted of two transistors (i.e., TFT1 and TFT2 described in Section 3.2); their structure and fabrication are described in Figure 7a. TFT1 exhibits a near‐zero *V*_T_, a low saturation voltage (≈0.48 V) and high gain (≈450). TFT2 as the depletion load has the same geometry as TFT1 under illumination. Consequently, some holes are generated and trapped at the gate insulator layer and interface between the dielectric layer and the IGZO. These trapped holes will produce free electrons in the channel layer, resulting in a negatively shifted *V*_T_. Thus, TFT‐2 has a bias current *I*_B_ of ≈90 pA at *V*_GS_ = 0 V, matching with TFT‐1 at *V*_GS_ = 0.51 V. During operation, when input voltage (*V*_in_) is less than 0.51 V, the output current (*I*_out_) is dominated by TFT‐1, increasing with *V*_in_. After *V*_in_ ≥ 0.51 V, *I*_out_ reach bias current of 90 pA (Figure 16c,e). A peak voltage gain ( *A*_V_ =  ∂*V*_out_/∂*V*_in_) of ≈220 is obtained at *V*_in_ = ≈0.5 V. Since the intrinsic gain (*A*_i_) for each TFT is 450, the peak voltage gain matches well with the relation of *A*_V_ = 0.5 *A*_i_. Moreover, the circuit shows a remarkably low output‐powder consumption (<150 pW), which is suitable for ultralow power circuits.

**Figure 17:**
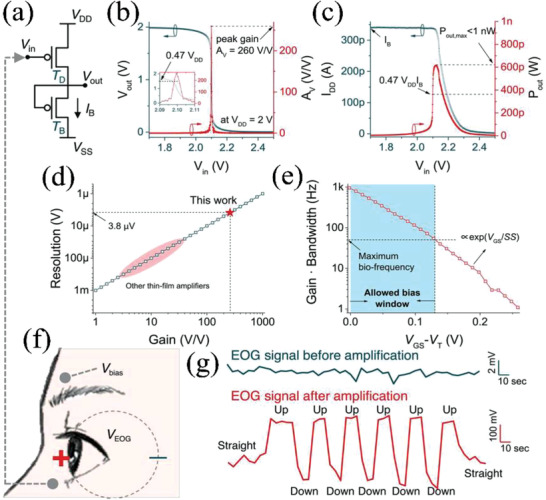
a) Circuit diagram for common‐source amplifier based on pairs of C8‐BTBT SB‐OTFTs in a common‐source configuration. b) Output voltage and gain of amplifier versus input voltage. c) Operating current and power consumption of amplifier circuit versus input voltage. d) Resolution for electrophysiological signal detection versus gain. e) Gain band width product of amplifier versus gate bias in the subthreshold regime. f) Operating principle and circuit configuration for EOG amplification with the amplifier. g) EOG signal before and after amplification. Reproduced with permission.^[^ ^88^ ^]^ Copyright 2019, American Association for the Advancement of Science.

The same research group also integrated amplifier circuits based on Schottky barrier (SB) OTFTs in a common‐source structure.^[^ ^88^ ^]^ Such SB‐OTFT used silver as the source/drain/gate electrodes, PVC as the gate dielectric, C8‐BTBT blended with PS as p‐type semiconductor, shown in Figure 12a. The circuit consists of drive and bias transistors (**Figure** **17**a), and exhibits steep variation in output voltage, and peak voltage gain of 260 (Figure 17b). Note, a higher gain of 290 was demonstrated with polysilicon SGTs by the Sporea group.^[^ ^93^ ^]^ For the subthreshold organic bias transistor, the power consumption was <1 nW due to low bias current of 342 pA (Figure 17c), which could be easily driven by low power source. This type of SB OTFTs amplifier is very promising for electrophysiological signal detection due to high resolution (<4 mV) compared with other thin‐film amplifiers (Figure 17d).

For electrophysiological signal analysis, the signal frequency should be limited to less than 50 Hz to screen out higher‐frequency neuronal activity. In this work, such an amplifier exhibited a large allowed bias window of 0.13 V for analog circuit design (Figure 17e). Based on these results, the amplifier was found to be suitable for human electro‐oculogram (EOG) signal detection, which refers to monitoring the corneo‐retinal potentials (*V*_EOG_). The potentials originating from the retinal pigment epithelium define two positions (Figure 17f): the front of the human eye (electrically positive) compared with the back (negative). Note that typical potentials amplitude ranges from 250 to 1000 µV with frequencies between 0 to 30 Hz. By employing the SB OTFT based amplifier, the amplitude of the amplified signal is higher than 0.3 V along with SNRs of > 60 dB. This amplifier offers enormous potential to monitor minimal eye movements for virtual and augmented reality. Thus, SGTs operate at low switching voltage (<5 V), ultralow power consumption (nW–µW) with high gain and high stability, properties that are suitable for ultralow‐power high‐gain amplifiers and provide enormous potential for wearable devices due to their limited battery capacity.

**Figure 18:**
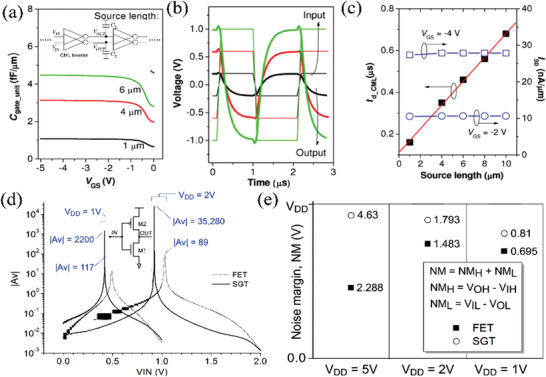
a) Unit gate capacitance (*C*_gate_unit_) of the OSGT versus different bias at different source length; inset: general configuration of two cascaded CML inverters based on pentacene SGTs. b) Input and simulated output signals of the first CML inverter at different signal swings. c) One‐stage delay and saturation current of CML inverter versus difference source length. a–c) Reproduced with permission.^[^ ^94^ ^]^ Copyright 2009, IEEE. d) Inverter gain based on polysilicon SGT and FET versus input voltages. e) Noise margin for SGT and FET inverters at different supply voltages. d,e) Reproduced with permission.^[^ ^95^ ^]^ Copyright 2014, Nature Publishing Group.

Shannon and co‐workers proposed high‐speed current‐mode logic (CML) circuits based on OSGTs.^[^ ^94^ ^]^ A 30 nm pentacene film was used as the semiconductor channel with a 50 nm thick SiO_2_ dielectric layer. These SGTs have a Schottky barrier height of 0.45 eV, a low saturation voltage (<2 V), and very flat output characteristics with a source–drain separation of 0.5 µm and source length of 1 µm. For the simulating a CML inverter based on two SGTs (insert, **Figure** **18**a), all transistors operate in the saturation state and a large gate swing is unnecessary to achieve efficient on/off switching. Therefore, the circuit speed is independent of the limited subthreshold characteristics. Moreover, the saturation current is unaffected by source–drain separation, which means imprecise and simple technologies for suitable mass‐production. The speed of inverter circuits is commonly characterized by a ring oscillator and thus two cascaded CML inverters can characterize the one‐stage delay (Figure 18a), and the one stage delay is characterized by first inverter transient simulations. This circuit delivers high performance under wide signal swings ranging from 0.4 to 2 V (Figure 18b), and the saturation current is invariant for different source lengths, meaning universality of such structure with source length under the same device parameters. The delay time (*t*_d___CML_) shows a nearly linear increase with increasing the source length, as *t*_d___CML_ is proportional to *C*_gate_, and *C*_gate_ has a linear relationship with the source length (Figure 18a). The delay time ranges from 0.16 to 0.64 µs, which is far shorter than the 1.67 µs in comparable OTFTs. The CML inverter based on OSGTs is much faster than a conventional full swing inverter and should be optimizable further using active loads. Therefore, SGT inverters can function with a wide range of signal swings and are very suitable for high‐speed electronics.

Additionally, Sporea and co‐workers fabricated complementary inverters based on polysilicon SGTs with a Schottky source contact and an ohmic drain contact.^[^ ^95^ ^]^ Through comparison between TFTs and SGTs, the authors reported that the SGT inverters exhibited 400 times higher gain than the TFT‐based inverters. The gain of the SGT inverter shows a lower spread, indicating a narrower switching region which benefits transient operation (Figure 18d). In addition, the SGT inverter provides a large enhancement in noise margin (Figure 18e, i.e., more than double at *V*_DD_ = 5 V and over 20% at *V*_DD_ = 2 V). The larger noise margin is significant for minimizing errors due to power supply glitches, electromagnetically coupled electrical noise, and extreme operating conditions, such as large fan‐out loads. In addition, the SGT inverter also has a slower transient current (17.5 fA s) versus 45 fA s for the TFT inverter. The authors suggested that these results are transferable to other logic gates, semiconductor materials, and process technologies.

## 5 Conclusions and Outlook

This review analyzes recent developments and attractions of unconventional materials for SGTs in terms of operating mechanism, promising materials, new device architectures, and real‐world applications. The presence of a source barrier exerts a significant effect on the creation of a depletion envelope beneath the source electrode, thus resulting in significant improvements in several key switching parameters. Using this device architecture also enables the utilization of several types of semiconducting materials ranging from inorganics, such as metal oxides, to 2D inorganic semiconductors, and organic semiconductors. This has enabled their integration and application in diverse high‐performance technologies such as photodetectors and inverter and logic circuits.

Since 2003, a number of studies focused on better understanding the operation mechanisms and performance of unconventional materials SGTs through varying the device geometry, channel layer, and various introduced interlayers. Remarkable improvements in performance have been achieved, such as lower saturation voltages, lower power dissipations, higher intrinsic gain, greater bias stress stability, and higher signal‐to‐noise ratios, versus traditional TFTs. Nevertheless, major challenges remain. Unconventional materials film deposition has been mostly limited to vapor deposition processes such as radio frequency sputtering and atomic layer deposition. These processes are expensive and time‐consuming due to the capital‐intensive vacuum‐based equipment, multiple‐step photolithography, and ultralong pump‐down cycles. Solution processes conducted at low temperatures have proven to be desirable for improving compatibility with flexible substrates and achieving low‐cost high‐throughput manufacture. Therefore, SGTs based on scalable and high‐quality semiconductors processed by solution methods (e.g., spin coating, inkjet‐printing, and blade coating) could become new potential research directions. Additionally, metal electrodes mostly adopted to achieve proper Schottky barriers have poor mechanical flexibility and optical transparency, which could be optimized through suitable electrode and semiconductor materials. Realization of mechanical flexibility and optical transparency in SGTs could also be an interesting research direction by incorporating oxide‐polymer hybrids and electrodes based on amorphous conducting oxides.^[^ ^96^ ^]^ Furthermore, SGTs exhibit a saturation current that is several orders lower than in conventional TFTs, which is not suitable for some applications such as switch mode power supplies, power electronic devices, low dropout regulators, etc. Thus, increases in current should be further pursued and optimized without significantly compromising other performance parameters. This issue may be addressed by geometrical adjustments in the device architecture, unconventional materials, and interfacial modification at the contacts. Expectantly, increasing channel width, doping the source region of the semiconductor and introducing tunnel layer or interlayer (heterostructure) between semiconductor and contact may contribute to higher current density.

SGTs will continue to be a promising area of fundamental research and development for several reasons. First, SGTs are more resistant to short‐channel effects and source/drain separation variations due to their unique operating mechanism. Therefore, device dimensions can be further scaled down to achieve high integration and miniaturization through specific and straightforward processing technologies. Second, the low supply voltage and low power consumption make SGTs as a promising candidate for wearable devices in Internet of Things due to limited battery capacity. Third, owing to the high intrinsic gain and stability, SGTs offer a huge potential in amplifiers and related circuits with high signal‐to‐noise ratios, implying high sensitivity to weak signals, making them ideal for implantable health care devices. Finally, the SGT operation mechanism and structure design disrupt the existing paradigm that only high‐performance semiconductors can serve as acceptable channel materials. SGTs show that excellent devices can be realized on a large scale with highly disordered and/or defective semiconductors. Therefore, a wide range of unconventional channel materials are likely suitable for SGTs, including highly conductive materials, poor‐quality semiconductors, and multifunctional materials. This could accelerate the development of fully integrated analog and digital circuits, and yield completely new types of devices. Based on this multitude of promising research directions, we believe that the recent emergence of unconventional semiconducting materials such as oxides, organics, and 2D materials, when combined with the aforementioned attractions of SGT architectures, offer great potential for emerging technologies and commercialization.

## Conflict of Interest

The authors declare no conflict of interest.
